# Integration of a laterally acquired gene into a cell network important for growth in a strain of *Vibrio rotiferianus*

**DOI:** 10.1186/1471-2180-11-253

**Published:** 2011-11-18

**Authors:** Maurizio Labbate, Yan Boucher, Piklu Roy Chowdhury, Hatch W Stokes

**Affiliations:** 1 The ithree institute, University of Technology, Sydney. Harris Street and Broadway, Sydney, NSW 2007, Australia; 2 Department of Biological Sciences, University of Alberta. 110 St NW Edmonton, Alberta, T6G 2R3, Canada

## Abstract

**Background:**

Lateral Gene Transfer (LGT) is a major contributor to bacterial evolution and up to 25% of a bacterium's genome may have been acquired by this process over evolutionary periods of time. Successful LGT requires both the physical transfer of DNA and its successful incorporation into the host cell. One system that contributes to this latter step by site-specific recombination is the integron. Integrons are found in many diverse bacterial Genera and is a genetic system ubiquitous in vibrios that captures mobile DNA at a dedicated site. The presence of integron-associated genes, contained within units of mobile DNA called gene cassettes makes up a substantial component of the vibrio genome (1-3%). Little is known about the role of this system since the vast majority of genes in vibrio arrays are highly novel and functions cannot be ascribed. It is generally regarded that strain-specific mobile genes cannot be readily integrated into the cellular machinery since any perturbation of core metabolism is likely to result in a loss of fitness.

**Results:**

In this study, at least one mobile gene contained within the *Vibrio rotiferianus *strain DAT722, but lacking close relatives elsewhere, is shown to greatly reduce host fitness when deleted and tested in growth assays. The precise role of the mobile gene product is unknown but impacts on the regulation of outermembrane porins. This demonstrates that strain specific laterally acquired mobile DNA can be integrated rapidly into bacterial networks such that it becomes advantageous for survival and adaptation in changing environments.

**Conclusions:**

Mobile genes that are highly strain specific are generally believed to act in isolation. This is because perturbation of existing cell machinery by the acquisition of a new gene by LGT is highly likely to lower fitness. In contrast, we show here that at least one mobile gene, apparently unique to a strain, encodes a product that has integrated into central cellular metabolic processes such that it greatly lowers fitness when lost under those conditions likely to be commonly encountered for the free living cell. This has ramifications for our understanding of the role mobile gene encoded products play in the cell from a systems biology perspective.

## Background

The integron includes a site-specific recombination system that integrates and expresses genes present on mobile elements called gene cassettes [1]. The integron platform is defined by three characteristics: an integrase gene (*intI*) whose product encodes a site-specific integrase, IntI, an attachment site (*attI*) at which point DNA sequences are inserted and a promoter (P_c_) which expresses genes within the gene cassettes inserted at *attI *[2]. Gene cassettes can be inserted into the integron as individual units but multiple events can lead to large tandem arrays. Integrons are best known for their role in the spread of antibiotic resistance genes in clinical environments [3]. These clinical integrons harbour 1-6 gene cassettes and are often associated with mobile elements such as resistance plasmids and transposons [3]. However, integrons are diverse genetic elements found in approximately 10% of environmental bacteria [2]. In these bacteria, integrons are found in chromosomal locations and rarely carry antibiotic resistance gene cassettes indicating a general role in evolution.

*Vibrio *is a genus of highly adaptable bacteria found in diverse marine-associated niches [4]. This adaptability is partly driven by lateral gene transfer (LGT), a process believed to be particularly important in this genus since the recent finding that *Vibrio cholerae *and other vibrios naturally take up DNA from the environment [5,6]. In the vibrio, integron cassette arrays can comprise well in excess of 100 cassettes [7]. Thus, the integron is a significant source of laterally acquired DNA in vibrio consisting of 1-3% of the total genome and generates genetic diversity even among closely related strains [2]. For example, it is the only identified genomic region that differs between some strains responsible for the current *V. cholerae *pandemic [8]. It has also been recently suggested that integron associated gene pools in the vibrios are important in adaptation to local environmental and ecological conditions [9].

Recent additional studies have provided new insight into the biology of vibrio integrons. The SOS stress response induces transcription of the integron-integrase increasing the rate of insertion, excision and shuffling of gene cassettes [10]. Furthermore, the majority of gene cassettes in a 116-cassette array [11] located in the *Vibrio rotiferianus *strain DAT722 [12] were found to be transcribed due to the presence of promoters distributed throughout the array [13]. Thus, cassette transcription is not absolutely dependent on being near P_c_. Collectively these findings suggest the integron provides a more prominent role in vibrio adaptation than previously thought.

Approximately 75% of integron associated gene cassette products in *Vibrio *species are novel with the remainder being designated with a putative function based on the presence of known domains through *in silico *analysis [2] or, to a very limited extent, by protein structural analysis [14]. The novelty of gene cassette products has made them difficult targets to study. However, like most mobile DNA, gene cassettes are believed to provide their host with accessory phenotypes imparting a niche-specific advantage. The exemplar of this phenomenon is antibiotic resistance, where most of the genes driving resistance adaptation are highly mobile [15]. This has also been supported by the handful of novel gene cassettes that have been characterised proving them to be functional and include genes potentially involved in pathogenesis in *V. cholerae *[14,16-18]. It is easy to understand how a protein carrying out a single biochemical reaction, for example the chemical inactivation of an antibiotic, can act in isolation and confer a strong selective advantage when the containing cell is in an environment where a toxic compound is present. This argument can also be extended to self contained sets of genes (operons) that encode pathways conferring resistance to such things as mercury and chromate which have also been captured and spread by mobilizing elements. It is largely believed that highly mobile genes would be confined to such function-types since laterally acquired genes that influence core metabolic functions are likely to lower fitness when first captured [19]. However we show here that at least one of eight novel cassettes associated with a vibrio integron encodes a product that is integrated into cell membrane porin regulation such that its loss would impact on cell fitness under physiological conditions that would normally be encountered by the free living host.

## Results

### Deletion of cassettes reduces growth on some carbon sources

**Figure 1:**
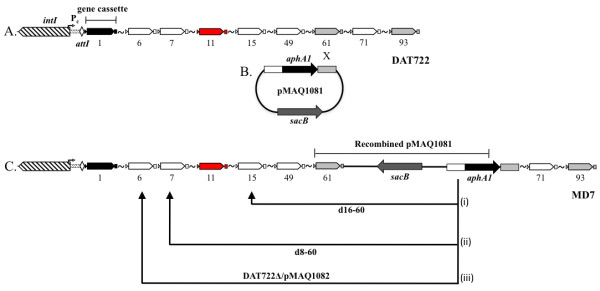
**Creation of deletions in cassette array of *Vibrio rotiferianus *DAT722**. Genetic features of the integron are labelled in the figure (A). The numbers below each cassette indicates its position in the array relative to *attI*. The white (group 1) and grey cassettes (group 2) indicate the two groups of paralogous cassettes described in the Materials and Methods section. Not all cassettes are shown with gaps of missing cassettes marked with a ~ symbol. A 1834 bp DNA fragment consisting of, in order, a portion of the white paralogous cassette sequence (448 bp), the *aphA1 *gene from pLOW2 (964 bp) and a portion of the grey paralogous sequence (410 bp) was cloned into the suicide vector pCVD442 to create pMAQ1081 (B). Plasmid pMAQ1081 was conjugated into *Vibrio *sp. DAT722-Sm resulting in a single crossover at cassette 61 creating strain MD7 (C). Counterselection of MD7 with sucrose medium resulted in isolation of deletion mutants that had undergone a second crossover with cassette 15, creating mutant d16-60 and deletion of cassettes 16 to 60 (C, i), with cassette 7 resulting in mutants d8-60a, d8-60b and d8-60c and deletion of cassettes 8 to 60 (C, ii).

**Figure 2:**
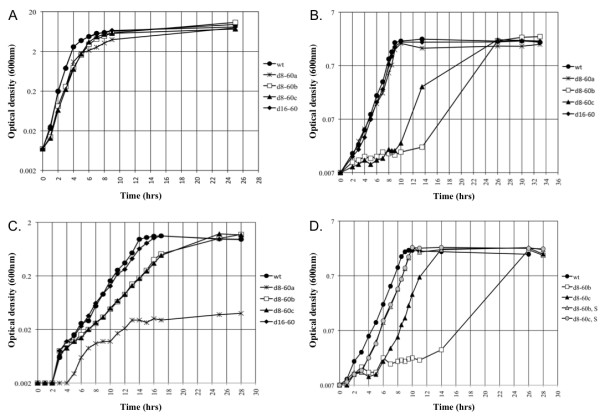
**Growth curves of *V. rotiferianus *DAT722-Sm (wt), d8-60 (d8-60a and d8-60b, d8-60c) and d16-60 deletion mutants in LB20 (A), 2M + glucose (B) and 2M + pyruvate (C)**. Growth curves of the spontaneous mutants d8-60b-S and d8-60c-S in 2M + glucose (D). Data presented are representative of results obtained in at least three independent experiments.

**Figure 3:**
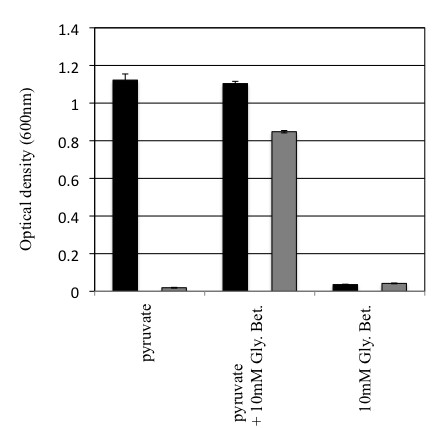
**Growth of d8-60a in 2M + pyruvate medium can be restored through the addition of osmoprotectant glycine-betaine (Gly. Bet)**. Final growth OD_600 _value of *V. rotiferianus *DAT722-sm (black bars) and the d8-60a mutant (grey bars) after 20 hours growth in 2M + pyruvate with and without glycine-betaine. As a control, pyruvate was removed from the medium as a carbon source to ensure glycine-betaine was not being used a carbon source.

To investigate how cassette genes may influence adaptation in their bacterial host, deletions of cassettes were created in the integron cassette array of *Vibrio rotiferianus *DAT722. Two cassette deletion mutants within the 116-gene cassette array of *Vibrio rotiferianus *DAT722 were created (See Methods and Figure 1). These mutants removed cassettes 8-60 (designated d8-60a) in one case and cassettes 16-60 (d16-60) in the other. The ability of these mutants to grow in various media were tested and compared to the wild type parent (Figure 2). It was found that both mutants were able to grow normally in a complete medium (LB20) albeit with a slightly extended lag phase for d8-60a (Figure 2A). The two mutants were also examined for growth in marine minimal medium (2M salts, a medium that mimics marine seawater [20]) with glucose (Figure 2B) or pyruvate (Figure 2C) as a sole carbon source. The growth of both mutants was normal compared to wild type (*Vibrio rotiferianus *DAT722) in 2M + glucose as was also the case for d16-60 in pyruvate. In contrast, d8-60a grew very poorly with pyruvate as sole carbon source. Growth of this mutant however could be restored on pyruvate with the addition of glycine-betaine, a known osmoprotectant (Figure 3). Glucose is also known to be a better osmoprotectant than pyruvate and we therefore tentatively conclude that the poor growth of d8-60a in pyruvate is a result of intolerance to osmotic changes and not a failure to extract carbon from this molecule. Further growth experiments supported this hypothesis with growth on other carbon sources that osmoprotect (eg trehalose) compared to failure to grow on other non-osmoprotectants (aspartic acid, glutamic acid, succinate and fumarate) (data not shown). These data suggested that this cassette array may include encoded proteins that integrate into and influence cellular processes more broadly in contrast to possessing proteins involved in single step secondary metabolism. Specifically, in DAT722, at least one cassette product appears to influence normal growth under nutrient conditions analogous to those found in seawater, the natural free-living environment for *Vibrio rotiferianus*.

To confirm that the dramatic reduction in fitness of d8-60a was a result of the loss of a mobile cassette and not the consequence of a spontaneous mutation elsewhere in the genome of the isolate selected for analysis, two other independent mutants, d8-60b and d8-60c, comprising loss of the same cassettes were constructed and examined for their growth characteristics. The results for these two mutants showed significant growth impairment in minimal medium although not in a manner identical to d8-60a. In glucose, both d8-60b and d8-60c had significant lag phases of up to 14 hours compared to wild type DAT722 and d8-60a but thereafter grew to achieve wild type cell densities at 24 hours (Figure 2B). In pyruvate, d8-60b and d8-60c showed reduced growth rates compared to DAT722 although they were significantly better than d8-60a (Figure 2C).

**Figure 4:**
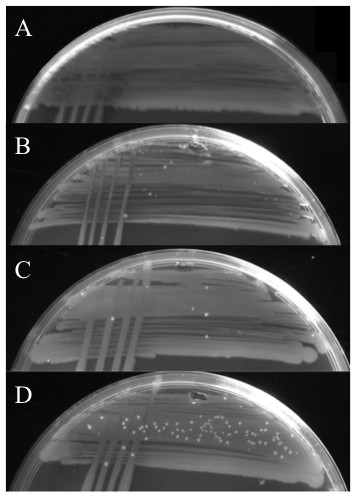
**Comparison of *V. rotiferianus *DAT722-Sm (A) and mutants d8-60a (B), d8-60b (C) and d8-60c (D) streaked on LB20 agar**. The d8-60 mutants show the presence of microcolonies on the streak line.

All three d8-60 mutants generated a minority of microcolonies when streaked on LB20 complete medium (Figure 4). This suggested that the mutants had an overall reduced fitness that was strongly selective for mutants that compensated for loss of a function encoded within the region deleted. The nature of these compensating mutations may thus explain the variability of growth seen between mutants in minimal medium. In support of the notion that compensating mutations were being selected out was the observation that cells recovered from microcolonies that showed enhanced growth showed wild type equivalent growth in minimal medium + glucose. This is shown in cell lines d8-60b-S and d8-60c-S in Figure 2D. Taking these data together we suggest that an integron associated cassette product participates in some aspect of cell metabolism that directly or indirectly impacts on growth such that a secondary mutation(s) is required to maintain viability or growth. This product must be encoded by one of the genes located in cassettes 8 to 15 inclusive since the smaller deletion encompassing cassettes 16-60 does not display any of these effects (Figure 2).

### Cassette deletions change the outermembrane protein profiles of cells

**Figure 5:**
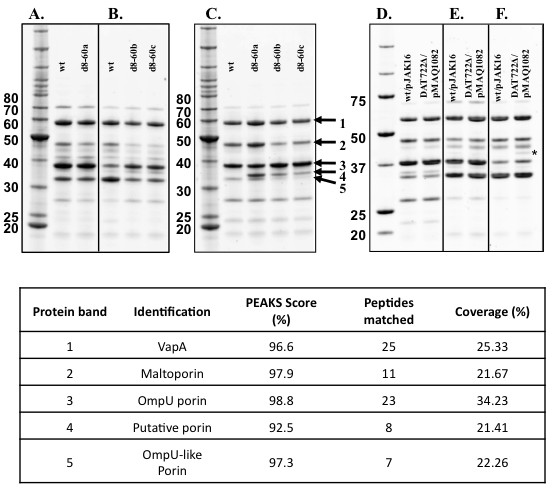
**Outermembrane protein (OMP) analysis of *V. rotiferianus *DAT722-Sm (wt) and d8-60 mutants grown in 2M + glucose (A), 2M + pyruvate (B) and 2M + LB nutrients (C)**. Labelled proteins in C were identified as 1) VapA, 2) Maltoporin, 3) OmpU porin, 4) putative porin and 5) OmpU-like porin as indicated in the Table below the panels. The molecular weight marker is given in the left most lane for panels A/B, C and D/E/F with the relevant sizes (in kDa) given left of the respective panels.

Porins play a major role in controlling the permeability of the outermembrane of Gram-negative bacteria. Changes in porin composition affect the cell's osmotic balance and nutrient transport [21]. Therefore, it was hypothesized that the likely osmotic shock of d8-60a in 2M + pyruvate and the growth defects of d8-60b and d8-60c in 2M + glucose might be due to changes in the composition of outermembrane porins. Outermembrane protein profiles showed significant changes in the composition of porins in all three d8-60 mutants compared to the wild-type using different growth media indicating an inability of these mutants to regulate their porins normally (Figure 5A, B and 5C). In 2M + glucose conditions, d8-60a showed slight decreases in four proteins identified as VapA (the structural subunit of a two-dimensional lattice in the outer membrane called the S-layer; band 1), maltoporin (band 2), OmpU porin (band 3) and an OmpU-like porin (band 4) compared to the wild-type, consistent with the healthy growth of d8-60a in this medium (Figure 5A). However, the changes in regulation of porins in d8-60a was clearly observed when grown in 2M + LB nutrients as it showed increased amounts of VapA (band 1) and maltoporin (band 2) and the presence of a putative porin (band 4) not detected in the wild-type under these nutrient conditions (Figure 5C). This irregular regulation explained the inability for d8-60a to grow in 2M salts without the presence of an osmoprotectant such as glycine-betaine or glucose to restore the osmotic balance.

The mutants d8-60b and d8-60c had very similar porin profiles, a result consistent with the similar growth phenotypes displayed by these mutants. In 2M + pyruvate conditions, a significant down-regulation of the maltoporin (band 2) and the OmpU-like porin (band 5) but an up-regulation of OmpU (band 3) was observed when compared to the wild-type (Figure 5B). In 2M + LB nutrient medium, these mutants had reduced levels of the maltoporin (band 2) and the presence of the putative porin (band 4) protein in replacement of the OmpU-like porin (band 5) compared to the wild-type (Figure 5C).

### Expression of a single gene cassette *in trans *maintains normal growth after generation of strains with deleted cassettes

Since mutant d16-60 (cassettes 16 to 60 deleted) had normal growth phenotypes compared to the wild-type, at least one cassette gene located between cassettes 7 and 16 has a strong pleiotropic affect. All eight cassettes within this region, except cassette 11, encode small hypothetical proteins with homology only to other cassette proteins. Therefore, nothing could be inferred regarding their putative function. However, cassette 11 includes a gene, encoding a 257 amino acid protein with pfam http://pfam.sanger.ac.uk/ identifying two domains; 1) an uncharacterized NERD domain at residues 31-150 that has weak homology to nucleases and is commonly associated with other protein domains involved in DNA processing [22], 2) a DNA-binding C4-zinc finger domain at residues 216-257 found in topoisomerase I proteins and involved in removing excessive negative supercoils from DNA [23]. Based on this bioinformatics analysis one possible biochemical function of the cassette 11 gene product is as a DNA topoisomerase. In addition, experiments with a mutated topoisomerase I (*topA*) gene have described phenotypes that are similar to those observed in the d8-60 mutants. Most notably, in characterized *topA *mutants, this includes the requirement for a compensatory mutation, emergence of spontaneous mutants and alterations in the composition of outermembrane porin proteins [23-28].

**Figure 6:**
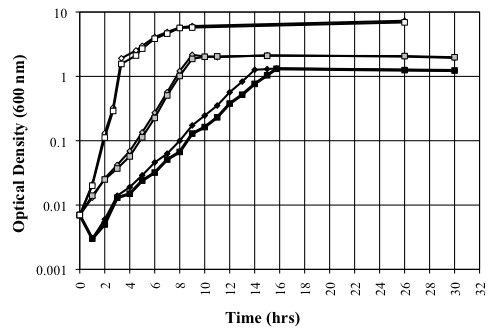
**Growth curves of *V. rotiferianus *DAT722-Sm/pJAK16 (squares) and DAT722Δ/pMAQ1082 (triangles) in LB20 (white), 2M + glucose (grey) and 2M + pyruvate (black)**. Data presented are representative of results obtained in three independent experiments.

To test for the cassette 11 gene product being responsible for the phenotype of the mutants described above, the plasmid pMAQ1082 was constructed which comprises only this cassette gene cloned into the vector pJAK16 (Methods). pMAQ1082 was then transformed into the merodiploid strain MD7. MD7 has a complete DAT722 cassette array and is the strain that was used to create the original deletion mutants (Methods and Figure 1). MD7/pMAQ1082 possesses a phenotype identical to that of DAT722 with respect to porin profiles and growth in LB20 and 2M media. From this strain, a deletion mutant was created, DAT722Δ/pMAQ1082 with the same cassettes deleted as strains d8-60a, b and c. The strain DAT722Δ/pMAQ1082 had no major growth defect (Figure 6) and possessed a wild type outermembrane protein profile in all tested media (Figure 5D, E, F). A slight decrease in growth rate was observed in 2M + pyruvate (Figure 6), which may be explained by the up-regulation of a protein (Figure 4F; marked with an asterisk) that is likely due to cassette 11 being removed from its native promoter.

## Discussion

The integron/gene cassette system is broadly dispersed amongst the Proteobacteria and is found in about 10% of sequenced genomes [2]. In the vibrios it is ubiquitous with arrays generally being especially large. Despite the fact that the integron gene cassette "metagenome" pool is very large [29,30], little is known about what the encoded proteins do beyond the enormous contribution some cassette proteins make to the antibiotic resistance problem [31]. A conventional understanding of cell metabolism would suggest they encode accessory phenotypes providing their host with a niche-specific advantage. Antibiotic resistance is a classic example of this since cassettes containing antibiotic resistance genes quite clearly provide a selective advantage in clinical environments where antibiotics are frequently used [31]. These highly mobilized genes frequently cross phylogenetic boundaries and a single gene can protect a cell from toxic compounds irrespective of the metabolic context in which it finds itself. The same phenomenon can extend to some adaptive genes that are part of a "self contained" unit as is the case, for example, in operons on transposons that confer mercury resistance [32].

The vibrios represent a diverse group of marine organisms and members of this group have very large cassette arrays. A typical vibrio cassette array comprises more than 100 novel genes [7]. Moreover, they represent the most dynamic component of the genome. In *V. cholerae*, pandemic strains that are otherwise indistinguishable by most phylogenetic typing techniques can still have very disparate cassette arrays [8]. Similarly, this is true for enclosed symbiotic communities of vibrios [33]. This highly mobile pool of genes, in a metagenomic sense, therefore number in at least the thousands and probably orders of magnitude more [29]. What do all these genes do? Many probably comprise functions that are metabolically independent of the rest of the cell in a manner analogous to antibiotic and heavy metal resistance genes. However, we show for the first time, that at least one mobile gene product can influence other aspects of core cell metabolism. In DAT722 this influence is such that at least one gene within the deleted region is highly adapted to this cell line to the extent that its loss reduces fitness to the point where the host cell is barely viable. The target gene or genes was contained to within a contiguous set of eight cassettes within the DAT722 array. Each of these cassettes contained a single predicted protein (Figure 1 and [11]). All of the predicted proteins are novel in that identical proteins are not present in any other known bacterium. Further, seven of the eight predicted proteins are highly novel to the point where they can only be described as hypothetical proteins. The remaining predicted protein, derived from cassette 11, is also novel although it contains a domain related to the DNA topoisomerase I family of proteins.

Although the precise function of this cassette protein needs to be established experimentally, the data generated was consistent with the hypothesis that the cassette 11 gene product was integrated into an essential cell network in the wild type DAT722. In particular, the fact that supplying this product alone *in trans *via pMAQ1082 preserved the wild type phenotype after subsequent deletion of cassettes 8 - 16 unambiguously points to an essential role in the cell porin regulatory network.

## Conclusions

Overall, this study emphasizes the importance of LGT in bacterial evolution and that this process can bring rapid adaptation not only through acquisition of novel functional genes, but more importantly through gain of genes that alter a cell's regulatory network. Thus, mobile genes can be adaptive over very short time scales such that their loss can threaten the viability of the cell through the disruption of a core metabolic process. This is in contrast to the generally held view that mobile DNA contributes to cell fitness by providing additional protein/s that act largely independently of core cell networks. Also, this data reinforces the point that large integron arrays are not solely dependent on P_c _for transcription since this cluster of genes if relatively distal to this promoter. It is clear therefore that despite the enormous increase in genomics and proteomic data in recent years, much is still to be learnt about the full of gamut of proteins necessary for important cell metabolic processes.

## Methods

### Strains, growth conditions and DNA purification

Bacterial strains and plasmids used in this study are listed in Table 1. Vibrio strains were routinely grown on Luria-Bertani medium supplemented with 2% NaCl (LB20). *Escherichia coli *strains were routinely grown on Luria-Bertani medium. Growth curves of all vibrio strains were conducted in 100 ml flasks containing 25 ml of medium. The inoculum was from overnight cultures grown in LB20 and then diluted to OD_600 _of 0.7 using 2% NaCl. Growth curve cultures were inoculated at 1:100. In experiments comparing growth of the wild-type and deletion mutants with different carbon sources, a marine minimal salts medium (2M) which mimics a seawater environment [20] was used supplemented with a carbon source (glucose and pyruvate at 11.1 mM and 20 mM respectively). Since growth of the d8-60 mutants in 2M was dependent on the added carbon source, 2M supplemented with LB nutrients (10 g tryptone and 5 g yeast extract per litre) was used to compare the outermembrane protein profiles of all mutants. In vibrio, kanamycin, chloramphenicol and streptomycin were used at 100 μg/ml, 12.5 μg/ml and 25 μg/ml respectively. In *E. coli*, kanamycin, chloramphenicol and ampicillin were used at concentrations of 50 μg/ml, 25 μg/ml and 100 μg/ml.

**Table 1:** List of strains and plasmids

Strain or plasmid	**Relevant genotype**^**a**^	Reference or source
Strains		
*V. rotiferianus *DAT722		
DAT722	Wild-type	[11]
DAT722-Sm	DAT722; Spontaneous Sm^R ^mutant.	This study
MD7	DAT722-Sm; Single recombination cross-over of pVSD2 into cassette 61, Km^R^	This study
d8-60a	DAT722-Sm; Δcassettes 8-60, Sm^R^, Km^R^	This study
d8-60b	DAT722-Sm; Δcassettes 8-60, Sm^R^, Km^R^	This study
d8-60b-S	DAT722-Sm; Δcassettes 8-60, Sm^R^, Km^R^. Spontaneous mutant of d8-60b.	This study
d8-60c	DAT722-Sm; Δcassettes 8-60, Sm^R^, Km^R^	This study
d8-60c-S	DAT722-Sm; Δcassettes 8-60, Sm^R^, Km^R^. Spontaneous mutant of d8-60c.	This study
d16-60	DAT722-Sm; Δcassettes 16-60, Sm^R^, Km^R^	This study
*E. coli*		
XL1-Blue	F' *proAB lacI^q^Z*ΔM15 Tn*10/recA1 endA1 gyrA96 thi-1 hsdR17 supE44 relAi*, Tc^R^	Stratagene
SY327 λ *pir*	Δ(*lac pro*) *argE *(Am) *rif nalA recA56*	[38]
SM10 λ *pir*	*thi thr leu tonA lacY supE recA*::RP4-2-Tc::Mu, Tc^r ^Km^R^	[39]
Plasmids		
pLOW2	Cloning vector, Km^R^	[40]
pGEM-T Easy	Cloning vector, Ap^R^	Promega
pMAQ1080	pGEM-T Easy carrying a 1834-bp fragment. The fragment was created using fusion PCR and consists of, in order, a 448-bp of paralog group 1 sequence, a 964-bp fragment containing *aphA1 *and a 410-bp paralog group 2 sequence abutted by *sal*I restriction sites.	This study
pCVD442	Mobilisable *sacB *counter-selectable suicide vector, Ap^R^	[41]
RK600 pJAK16 pMAQ1081 pMAQ1082	ColE1 *oriV*; RP4*tra^+ ^*RP4 *oriT*; Cm^R^; helper plasmid in triparental matings Low copy IPTG-inducible expression vector, Cm^R^ *sal*I fragment from pMAQ1080 cloned into the unique *sal*I site of pCVD442. pJAK16 containing cassette 11	[42] [43] This study This study

^a^Sm^R^, streptomycin resistance; Km^R^, kanamycin resistance; Tc^R^, tetracycline resistance; Ap^R^, Ampicillin resistance

*V. rotiferianus *DAT722 was isolated from a mud crab aquaculture tank in Darwin (Northern Territory, Australia) [11]. It was typed by multi locus sequence analysis of the *recA*, *pyrH*, *rpoA, topA, ftsZ *and *mreB *genes (data not shown). Transformation of *E. coli *XL1-Blue was performed as previously described [34]. Genomic DNA (gDNA) was extracted from overnight cultures using the Purelink genomic DNA mini kit (Invitrogen). Standard PCR was performed using high fidelity platinum *Taq *(Invitrogen) as per the manufacturer's instructions. Primers (Table 2) were used at a final concentration of 0.5 μM each. Plasmid pMAQ1082 was created by amplifying the cassette 11 gene from *V. rotiferianus *DAT722 using primers B-VSD11-F and P-VSD11-R (Table 2). The resulting amplicon was directionally cloned in front of the *lac *promoter using *BamH*I and *Pst*I. pMAQ1082 was conjugated into MD7 in a triparental conjugation using RK600 as the helper strain.

**Table 2:** Primers used in this study

Primer	Sequence (5'-3')	Target
PRG1-F	GTC GAC CAA AAT TTG GCT GCT TGT TG	Paralog 1 gene cassettes in *Vibrio rotiferianus *DAT722
PRG1-R	CAT CAG AGA TTT TGA GAC ACA ACC CGA GCG ACA ATT TTA AGC	Paralog 1 gene cassettes in *Vibrio rotiferianus *DAT722
PRG5-F	GGC AGA GCA TTA CGC TGA TCA AAG GTC ATA AGT TTT GGT G	Paralog 2 gene cassettes in *Vibrio rotiferianus *DAT722
PRG5-R	GTC GAC CAT GCG CTA CTT CTA TTT ATG C	Paralog 2 gene cassettes in *Vibrio rotiferianus *DAT722
Kan-F	GTT GTG TCT CAA AAT CTC TGA TG	*aphA1 *in pLOW2 (F)
Kan-R	TCA GCG TAA TGC TCT GCC	*aphA1 *in pLOW2 (R)
VSD5-F	TGA GCT ACC ACA AGC AAG G	Cassette 5 in *Vibrio rotiferianus *DAT722 (F)
VSD14-F	AAA GCG GTT ACA TTC GGG	Cassette 5 in *Vibrio rotiferianus *DAT722 (R)
VSD25a-F	ACA TAT GTA GAC CCT GTG CG	Cassette 25 in *Vibrio rotiferianus *DAT722 (F)
VSD47-F	CAT TTT AAG TCG GCT CTT CC	Cassette 25 in *Vibrio rotiferianus *DAT722 (R)
VSD62-R	GTA GGT AAT TTC GGC TTC TCG	Cassette 62 in *Vibrio rotiferianus *DAT722 (R)
VSD25b-F	TGC GCA ATA TAT CGC AAG AG	Cassette 25 in *Vibrio rotiferianus *DAT722 (F)
VSD25-R	GCC GTC CAT AGT CGT CAT TT	Cassette 25 in *Vibrio rotiferianus *sp. DAT722 (R)
B-VSD11-F	TTT TGG ATC CGA ATA GGG AAA ATC CGT G	Gene from cassette 11 in *V. rotiferianus *DAT722 (F)
P-VSD11-R	TTT TCT GCA GTT AGT TGA ATT GTT TCA CAG C	Gene from cassette 11 in *V. rotiferianus *DAT722 (R)

### DAT722 cassette analysis and strain construction

The cassette array of DAT722 is fully sequenced [12] and consists of 116 gene cassettes although there are 94 different cassette types due to the presence of paralogous cassettes [11]. For the deletion of cassettes by homologous recombination, the presence of paralogous cassettes in different positions of the array was exploited. Two of the paralogous cassette types were selected based on their position in the array. The first paralogous cassette type (group 1) is in positions 6, 7, 15, 27, 49, 66, 71, 76, 77 and 111. The second paralogous group (group 2) is in positions 34, 61, 83, 87, 90, 93 and 105. Using fusion PCR, a 1834 bp DNA fragment consisting of, in order, a portion of group 1 sequence (448 bp), the *aphA1 *gene from pLOW2 (964 bp) and a portion of group 2 sequence (410 bp) was amplified and cloned into pGEM-T Easy producing pMAQ1080. The fragment was excised from pMAQ1080 using *sal*I and cloned into the *sal*I site of the *sacB*-counter selectable suicide vector pCVD442 to create pMAQ1081. Homologous recombination (allele replacement) was used to replace cassettes between group 1 and group 2 cassettes with the 1834 bp fragment created by fusion PCR. Plasmid pMAQ1081 was conjugated into DAT722-Sm using *E. coli *SM10 as a donor with recombinants selected on LB20 medium supplemented with 100 μg/ml and 25 μg/ml of kanamycin and streptomycin respectively. A merodiploid (designated MD7) was isolated with pMAQ1081 recombining into cassette 61 of the integron cassette array (see Figure 1). An overnight culture of MD7 was inoculated into fresh LB20 at a dilution of 10^-6 ^and grown until turbidity was evident (~ 6 hours). For selection of double cross-over recombinants, a dilution series of the MD7 culture was plated onto LB medium containing 0.4% NaCl, 10% sucrose and 100 μg/ml kanamycin. Using primers targeting unique cassettes outside the expected deletions (Table 2), colonies were screened for the presence of deletions between 6/7 and 61, 15 and 61, 27 and 61 and 49 and 61. In the case of the mutants d8-60a, d8-60b, d8-60c, all three generated identical length PCR products by this method indicating identical deletion end points.

### Membrane protein analysis

The outer membrane proteins (OMPs) were extracted as previously described [35] using equal number of cells (equivalent to 5 ml of cells diluted to an OD_600 _of 5.0). The membrane pellet was resuspended in 200 μl of SDS sample buffer containing 5 mM tributylphosphine and 20 mM acrylamide for reduction and alkylation of proteins [36]. The solubilized proteins were diluted 1:5 in SDS sample buffer and 5 μl subject to polyacrylamide gel electrophoresis using a Criterion XT precast gel (4-12% Bis-Tris; Bio-Rad). Protein gels were stained with Flamingo protein stain (Bio-Rad) and imaged using a Pharos FX Plus Molecular Imager (Bio-Rad). Flamingo stained protein gels were post-stained with colloidal Coomassie G-250 stain and proteins of interest excised for identification by LC-MS/MS as previously described [37]. PEAKS software (Bioinformatics Solutions Inc.) was used to directly search peptides against a protein sequence FASTA output derived from the *V. rotiferianus *DAT722 genome [12]. The highest PEAKS score (percentage based on a p-value < 0.05) was taken as the closest peptide match. The full sequence of identified proteins is given in the additional file 1.

## Authors' contributions

ML and HWS designed the research; ML and PR performed the research; ML and YB analyzed data; ML and HWS wrote the paper. All authors have read and approved the final manuscript.

## Supplementary Material

Additional file 1 **lists the full sequence of outermembrane proteins that showed changes in concentration between wild type DAT722 and the mutant d8-60a under particular growth conditions**. Proteins were identified via LC-MS/MS analysis as described in the methods. Click here for file
