# Supplementary material for: Integration of a laterally acquired gene into a cell network important for growth in a strain of Vibrio rotiferianus
Source: BMC Microbiol. 2011 Nov 18;11:253. doi: 10.1186/1471-2180-11-253 (PMC3262767; doi:10.1186/1471-2180-11-253)
Supplement: Additional file 1 — lists the full sequence of outermembrane proteins that showed changes in concentration between wild type DAT722 and the mutant d8-60a under particular growth conditions. Proteins were identified via LC-MS/MS analysis as described in the methods. [file 1471-2180-11-253-S1.DOC]

>VapA
MLKKTLLATAVALASTSAFAAPEWSLTLNTNAVAPNNQFETNVLAVQTKQPVLDKTSLDAIATLVFDNSTAGDLALEDAGEIVLTLNGDAEFNADKVAALLTASSAAGAPAVFNGGLLVNIADDDDNLAYAATTFNPDGTVNVAGADSVEHIKKVFKVDEGAQSVAIRYNLDNGNKRLSIKLADDVSTAGTAVDKFVGVQFDFYNAVLKQAFKLTSGSVSSVSLEFGALKDATYSAEPKSKSVFKLEDMFKLTQTRAGLNSDGADKTALVASTYTQWDNPTLGGLRAVQFHNNTTNQQIYRSDVTIALSGDFAPIAKNGTSLVDAGGVNQPNWTLSDGVVSAKASAFNGGVFANLDDTPLELPSLFIPADNQVNIEAQKYALKVSHSGNATYNAYSQTIGDAFIVVRDGMKFDTVTTGTTSSNVIYIRDVSKTLPEDGGKIFVTITEYAEHANDAQGAGEDVVVRKELSTRLPSNGAVTLTPVGIAADLGVELNPARQARFFFEVETNQGEAAVKKQTADGVDIQTGDKAAPVDFTL

>Maltoporin

MKKVSAIAAAVAATLAAGSAFAVDFHGYMRAGVGMNADGGQQVSFEKNKLGRLGNEDDVYGEIQLGKEVYNNDGKTFYVDTMFAMTSNGSNDWEATEKDDAKFALRQFNVQAKGVLDFAPEATLWAGKRYYQRHDIHATDFYYWDMSGAGAGIENIEAGAGKLSVAWVRNDRNDTPPWNPEGDTPDAGNNGGAVNVNTFDVRYAGLPVWDNGSLELGASYAMVNETDDASAGAKAAQDGVLLTAELTQGLDSGFNKTVFQYGTEGYSKTLAYYGGGSWYGAEAADGASGYRLINWGVIGMGENWEMMHQLAYSVGEDMWNGMDKHESISAVVRPVYKWDDNHKTIFEAGYAIDDTDGAENKYGKLTVAQAWSAGSSFWARPEIRLYASYLTADKDDNTNAFDGGRSDDTFQFGVQAEAWW

>OmpU Porin

MKKTLIALSVSAAAMATGVNAAELYNQDGTSLEMGGRAEARLSMKDGDVADKSRIRLNFLGTQAINDNLYGVGFWEGEFTTSDEGKVDSNSSDLTTRYAYAGLGGAFGEVTYGKNDGALGVITDFTDIMAYAGNSAADKLAVADRADNMLAYKGQFENLAVKASYRFADRDIQNENTPNAEYTDNGEDGYSLSAIYAFADTGLELGAGYADQDEANEYMLAASYTMGDLYFAGVFTDGEKAKKDGDYTGYELAGAYTLGQTVFTSTYNNAETNNETSANNFAVDASYYFKPNFRGYVSYNFNLIDAGDKLGQVHGNTIASKVDAEDELALGLRYDF

>Putative Porin

MKMKTLAVAVAALACGSQAFAAEVYNSDGTSMSIGGHFTTDIGDNGDNVEVGEVSPRINIVGKQDLGNGLTVEAKGEWQTNLVDGGDTTFSTRLGYLALHSEMGKVTVGTQWSPYYSVASLTDIPIKYANDFLYDGGRSGSWRSIGTGRANKMLSYSNNFDFGNDMSLYFGVGWQGETKAGGSQFDDRGQIAASLSFSDFNVGLAYTGGDVDSEKLTSTAIAASYGSYGKGIYVAGVYGLNENLYGMTDSDQISGLLAYGLDNGLNFILYYENVDRDDNVLNPNRDVLAPQVEYSVTSKLKTFAGFRTHMGDDKSDDEWSLGARYYF

>OmpU-like Porin

MKKAALSTAVLTALVSAPSFAATVYDNEGTTLKVGGRAEARFNISDKHEKDGNSSFKDKSRARVNLKGKSQISDDLYGFGKYEAEFDDSSDITNRYFFAGLGTSIGEFSYGKQDSAQVMLTDITDTMATFGAEAADIVAGNKDKRENNFLYSGEFDALTIKANFIASDAKDKDSFGLAGLYNFGAFDIGAGYVSQSNGDNDDDQFNLVGQYSMDAFTLGALFTIGTVADDDYTGYELSAIYKPMKNLSLVGVYNFQEVDPKAGSKEDTVDEFAIEAVYKFNGHLRTYAGYKFQQIDSGDDELQAGIRYDF
